# Pain Relief Improves Key Motor Functions in Refractory Advanced Knee Osteoarthritis

**DOI:** 10.7759/cureus.83944

**Published:** 2025-05-12

**Authors:** Yoshinori Ishii, Hideo Noguchi, Junko Sato, Ikuko Takahashi, Hana Ishii, Ryo Ishii, Kei Ishii, Kai Ishii, Shin-ichi Toyabe, Taishi Ogawara

**Affiliations:** 1 Orthopaedic Surgery, Ishii Orthopaedic and Rehabilitation Clinic, Gyoda, JPN; 2 Orthopaedics, Ishii Orthopaedic and Rehabilitation Clinic, Gyoda, JPN; 3 Plastic and Reconstructive Surgery, Ishii Orthopaedic and Rehabilitation Clinic, Gyoda, JPN; 4 Department of Orthopaedic Surgery, Shinshu University Hospital, Matsumoto, JPN; 5 Breast Surgery, Ishii Orthopaedic and Rehabilitation Clinic, Gyoda, JPN; 6 Division of Information Science and Biostatistics, Niigata University Hospital, Niigata, JPN; 7 Department of Rehabilitation Medicine, Ishii Orthopaedic and Rehabilitation Clinic, Gyoda, JPN

**Keywords:** advanced knee osteoarthritis, diclofenac-etalhyaluronate, motor function, pain management, quadriceps femoris strength, range of motion, single-legged-stance

## Abstract

Background: Advanced knee osteoarthritis (KOA) severely impacts function and quality of life. While pain reduction is thought to improve motor function, the direct relationship remains unclear, especially in refractory cases. This study investigated the impact of pain relief following intra-articular diclofenac-etalhyaluronate (DF-HA) on motor function in advanced KOA patients unresponsive to conventional treatments.

Methods: This retrospective study included 100 knees (82 patients, Kellgren-Lawrence (KL) grade III/ IV) who received DF-HA. Patients were categorized into an Improved Group (IM-G, n= 88 knees) and a Non-Improved Group (Non-IM-G, n= 12 knees) based on visual analog scale (VAS) pain score changes 3-7 days post-injection. Japanese Orthopaedic Association Score for Osteoarthritis of the Knee (JOAS), range of motion (ROM), quadriceps femoris strength (QF), and single-legged stance (SLS) were assessed pre- and post-injection. Non-parametric tests compared changes within and between groups.

Results: In the IM-G, significant improvements were observed in JOAS (p< 0.001), ROM (p< 0.001), QF (p< 0.001), and SLS (p = 0.003). Conversely, the Non-IM-G showed no significant changes in JOAS, ROM, or QF, but a significant improvement in SLS (p = 0.024).

Conclusion: Aggressive pain management with DF-HA significantly improved key motor functions in advanced KOA patients experiencing pain relief. However, balance improvement may involve mechanisms beyond pain reduction. These findings highlight the importance of pain control in improving motor function in refractory KOA.

## Introduction

Knee osteoarthritis (KOA) is a prevalent condition that significantly diminishes the functional capacity and substantially impairs the quality of life (QOL) of affected individuals. Orthopedic surgeons employ a range of therapeutic strategies to manage KOA, guided by the Kellgren-Lawrence (KL) classification [1], encompassing lifestyle modifications through surgical interventions [2]. Common approaches include rehabilitation, intra-articular injections, and pharmacotherapy for early-stage KOA [3], as well as surgical management for advanced KOA [4].

Prior research suggests a potential link between pain reduction and improved motor function. For instance, Sun et al. [5] reported enhanced physical function, including balance, following pain relief after intra-articular hyaluronic acid injections in patients with mild KOA. Furthermore, in advanced KOA, total knee arthroplasty has been shown to result in increased range of motion (ROM) [6], quadriceps muscle volume and strength gains [7], and improved balance [8], indicating that pain alleviation achieved through surgery significantly contributes to the recovery of motor function. However, the precise mechanisms by which knee joint pain in KOA directly impairs knee motor function (ROM, muscle strength, balance) remain incompletely elucidated. Traditional conservative treatments, particularly oral and topical medications, often exhibit a delayed onset of action and significant inter-individual variability, making it challenging to establish an immediate correlation between pain reduction and improvements in motor function.

Against this backdrop, diclofenac-etalhyaluronate (DF-HA) (JOYCLU®, Ono Pharmaceutical Co., Ltd., Osaka, Japan), a novel intra-articular injection combining hyaluronic acid and diclofenac, has recently emerged as a new therapeutic option for KOA. DF-HA harnesses the potent anti-inflammatory and analgesic effects of diclofenac along with the joint function-improving properties of hyaluronic acid. Its sustained drug release and prolonged intra-articular retention are anticipated to yield synergistic therapeutic benefits. The proposed mechanisms of action include synovial fluid normalization, chondrodegeneration inhibition, and direct anti-inflammatory and analgesic effects [9, 10]. While previous studies have primarily utilized the Western Ontario and McMaster Universities Osteoarthritis Index (WOMAC) score [11] for evaluation, its clinical efficacy in both pain reduction and joint function improvement has garnered significant attention [12, 13].

Therefore, the objective of this study is to investigate the direct impact of pain reduction on motor function in patients with advanced KOA refractory to conventional conservative treatments. This will be achieved by administering intra-articular DF-HA and categorizing patients based on the presence or absence of pain improvement as assessed by the visual analog scale (VAS). Subsequently, we will conduct a detailed analysis of pre- and post-injection changes in knee joint ROM, periarticular muscle strength, and single-legged stance (SLS) between the two groups. The findings of this research have the potential to yield crucial insights for establishing novel treatment strategies for patients with refractory knee joint pain due to KOA. Furthermore, we firmly believe that this study holds significant importance in re-emphasizing to us, orthopedic surgeons, the indispensable role of pain management in improving motor function.

## Materials and methods

This study received approval (2020-10) from the IRB of the authors’ affiliated institutions. All patients received a thorough explanation regarding the DF-HA intra-articular injection procedure, anticipated benefits, and potential complications, and provided written informed consent prior to participation.

Study subjects

This retrospective study included 100 knees from 82 patients who underwent DF-HA intra-articular injections at our institution between May 2021 and March 2025. The inclusion criteria were a diagnosis of KOA with a KL grade of III or higher [1], and inadequate pain control despite prior intra-articular injections of hyaluronic acid or steroid preparations, or treatment with non-steroidal anti-inflammatory drugs (NSAIDs). Patients with a history of adverse reactions to hyaluronic acid or NSAIDs were excluded.

Patient group classification

**Figure 1:**
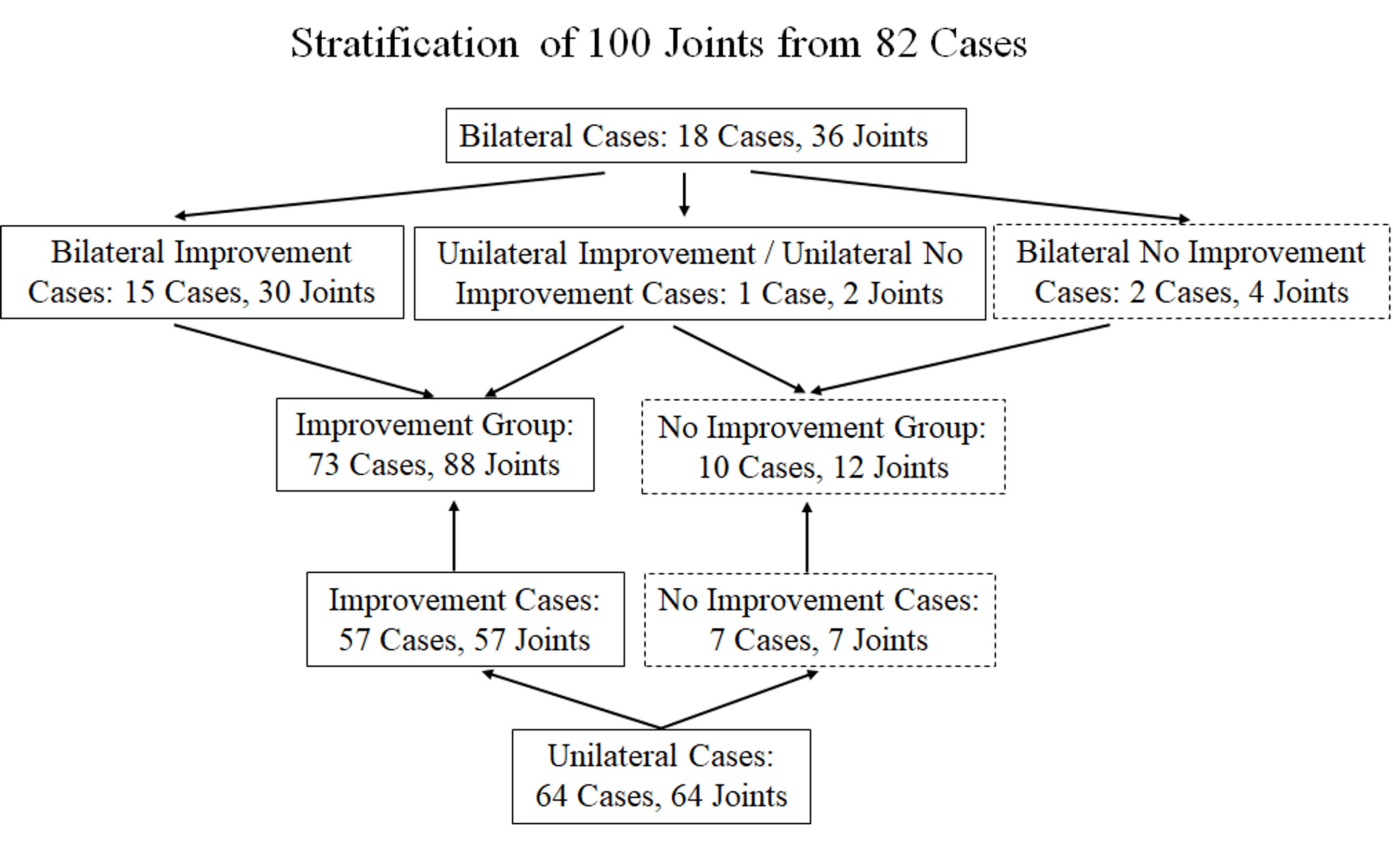
Stratification of 100 Knee Joints According to Clinical Response (Pain Relief) to Intra-articular Injection

Patients self-assessed their pain levels using the VAS score prior to DF-HA injection and between 3 and 7 days post-injection. Based on the change in VAS scores, the 88 joints (KL III 44, KL IV 44) exhibiting pain improvement were categorized into the Improved Group (IM-G), while the 12 joints (KL III 5, KL IV 7) showing no pain improvement were classified into the Non-Improved Group (Non-IM-G) (Figure 1). Further details are provided in Table 1.

**Table 1:** Patients’ characteristics Values presented as medians (interquartile range) {range} BL: body length; BW: body weight; BMI: body mass index

Variables	Improvement N= 73 patients, 88 knees	Non-improvement N=10 patients, 12 knees
Age	71 (63, 77) {49- 87}	72 (66, 81) {49- 87}
BL (cm)	156 (149, 163) {43- 180}	149 (147, 157) {143- 170}
BW (kg)	64 (55, 72) {43- 117}	64 (57, 69) {45- 117}
BMI (kg/m^2^)	26 (22, 29) {19- 45}	28 (25, 29) {21- 45}

Evaluation Items and Analysis Methods

To comprehensively evaluate the impact of intra-articular DF-HA injections on knee function in patients with KOA, the following parameters were measured by the attending physical therapist (PT).

Clinical Assessment

KL Grade [1]: Standing anteroposterior radiographs of both knees were obtained for all patients. These radiographs were graded according to the KL classification to determine the severity of osteoarthritis. The KL grade ranges from I to IV, with higher grades indicating more advanced disease.

Japanese Orthopaedic Association Score for Osteoarthritis of the Knee (JOAS): The validated and reliable JOAS [14] was utilized to objectively assess the functional status of patients with KOA. The JOAS comprises four domains: “Pain on walking” (30 points maximum), “Pain on stair climbing” (25 points maximum), “ROM” (35 points maximum), and “Swelling” (10 points maximum), yielding a total score of 100 points. A higher total score indicates better knee joint function.

Physiotherapy Assessment: Experienced PT Conducted the Following Assessments

ROM: With the patient in a supine position and under non-weight-bearing conditions, passive knee extension and flexion were performed, and ROM was measured using a standard 38 cm arm handheld goniometer. The axis of the goniometer was aligned with the lateral femoral epicondyle, the proximal arm with the greater trochanter, and the distal arm with the lateral malleolus. ROM was calculated as the difference between the maximum extension angle and the maximum flexion angle and recorded in 5° increments.

Muscle Strength: QF: Isometric knee extension strength was measured in Newtons (N) using a Locomo-Scan Dynamometer (Alcare Co., Ltd., Tokyo, Japan) with the knee joint positioned at approximately 20 ° of flexion (in accordance with Omori et al. [15]). Three measurements were taken on the affected side, and the highest value was used for analysis. To account for individual variations in body size, the measured value was divided by body weight (BW; kg), and the QF-to-BW ratio (QF/ BW ratio; N/ kg) was used for analysis.

Trunk Balance (Static Balance Ability): SLS test with eyes open: Static balance ability on the dominant leg was assessed. Patients were instructed to place both hands on their hips, fix their gaze on a target on the wall, and lift one leg, maintaining a standing position for as long as possible. The time until loss of balance or until reaching the maximum measurement time of 60 seconds was recorded. Two trials were performed on the affected side, and the longer duration, recorded to the nearest 0.1 second, was used for analysis.

Statistical analysis

Based on the results of Q-Q plots, the Kolmogorov-Smirnov test, and the Shapiro-Wilk test, the continuous variable data in this study were determined to be non-normally distributed. Consequently, the non-parametric Wilcoxon signed-rank test was employed for comparisons of paired continuous variables.

A post-hoc power analysis was conducted based on the sample size of this study, with the significance level set at α = 0.05. For the two-tailed Wilcoxon signed-rank test, the achieved power was 99.9% for the IM-G (N=88), which is very high, while it was 41.0% for the Non-IM-G (N=12), which is low. This result suggests that the smaller sample size in the Non-IM-G may have influenced statistical power.

All statistical analyses were performed using the statistical software R version 4.4.5 (R Foundation for Statistical Computing, Vienna, Austria) and G*Power version 3.1. A p-value of less than 0.05 was considered statistically significant. Data is presented as median and interquartile range (IQR) (25th percentile, 75th percentile).

## Results

We observed no incidence of serious adverse events attributable to the intra-articular DF-HA injections during the study period.

In the IM-G, intra-articular DF-HA injection resulted in significant improvements across multiple clinical evaluation parameters. The JOAS significantly increased from a pre-injection median of 65 points (IQR 60-70 points) to 73 points (IQR 65-85 points) post-injection (p< 0.001). Similarly, the ROM significantly expanded from a pre-injection median of 108° (IQR 95-120°) to 115° (IQR 100-121°) post-injection (p < 0.001). Regarding QF, the weight-normalized extension force significantly increased from a pre-injection median of 4.3 N/kg (IQR 2.7-5.6 N/kg) to 5.0 N/kg (IQR 4.0-6.2 N/kg) post-injection (p < 0.001). Furthermore, the SLS time significantly lengthened from a pre-injection median of 12.5 seconds (IQR 4.5-48.6 seconds) to 18.7 seconds (IQR 5.9-60.0 seconds) post-injection (p = 0.003) (Table 2).

**Table 2:** Changes in scores and joint function in pain improvement group (IM-G) Values presented as medians (interquartile range) DF-HA: diclofenac-etalhyaluronate, VAS: visual analog scale, JOAS: Japanese Orthopaedic Association Score, ROM: range of motion, QF: quadriceps femoris strength, SLS: single-legged stance

Variables (N=88)	Pre-DF-HA	Post-DF-HA	Differences	p-value
VAS score	6 (5, 8)	3 (2, 4)	3 (2, 4)	<0.001
JOAS	65 (60, 70)	73 (65, 85)	5 (0, 10)	<0.001
ROM (°)	108 (95, 120)	115 (100, 121)	5 (0, 10)	<0.001
QF (N/kg)	4.3 (2.7, 5.6)	5.0 (4.0, 6.2)	0.6 (-0.2, 1.8)	<0.001
SLS (minutes)	12.5 (4.5, 48.6)	18.7 (5.9, 60.0)	0.4 (0.0, 5.5)	0.003

Conversely, in the Non-IM-G, no significant changes were observed in the JOAS, ROM, or QF following intra-articular DF-HA injection (JOAS; p = 0.374, ROM; p = 0.834, QF; p = 0.339). However, SLS time was the only parameter that showed a significant increase, from a pre-injection median of 7.3 seconds (IQR 2.3-19.1 seconds) to 9.0 seconds (IQR 5.8-57.8 seconds) post-injection (p = 0.024) (Table 3).

**Table 3:** Changes in scores and joint function in pain non-improvement group (Non-IM-G) Values presented as medians (interquartile range) DF-HA: diclofenac-etalhyaluronate, VAS: visual analog scale, JOAS: Japanese Orthopaedic Association Score, ROM: range of motion, QF: quadriceps femoris strength, SLS: single-legged stance

Variables (N= 12)	Pre-DF-HA	Post-DF-HA	Differences	p
VAS score	6 (3, 8)	6 (3, 8)	0 (0, 0)	N/A
JOAS	60 (54, 68)	65 (50, 71)	5 (0, 5)	0.374
ROM (°)	113 (100, 125)	110 (109, 121)	0 (0, 6)	0.834
QF (N/kg)	3.6 (2.7, 5.5)	4.1 (2.7, 5.2)	0.3 (-0.5, 0.7)	0.339
SLS (minutes)	7.3 (2.3, 19.1)	9.0 (5.8, 57.8)	2.3 (0.0, 7.7)	0.024

## Discussion

A notable aspect of this study is its focus on patients with advanced KOA who had not responded to conventional conservative treatments. We specifically compared the impact of pain relief on motor function by clearly dividing the patients into two groups based on the presence or absence of pain improvement. The results demonstrated statistically significant improvements in the IM-G across all measured parameters: the JOAS, ROM, QF, and SLS time.

These findings strongly suggest that the alleviation of persistent pain in patients with advanced KOA, who have historically been challenging to treat, is not merely a reduction in subjective complaints but is also deeply associated with improvements in objective motor functions directly relevant to gait and activities of daily living. Notably, the significant increase in JOAS likely translates directly to enhanced daily living activities, potentially contributing substantially to improved patient QOL. Furthermore, the increased ROM and enhanced QF suggest improved knee joint stability and mobility, while the prolonged SLS time indicates an improvement in balance ability. Consistent with prior research [16, 17], chronic pain in advanced KOA is known to induce periarticular muscle atrophy and restricted joint mobility, consequently leading to diminished daily living activities and impaired balance. The improvements in motor function observed in this study following pain reduction provide clear clinical evidence supporting the notion that pain is a direct inhibitory factor on motor function.

Conversely, in the Non-IM-G, no significant changes were observed in the JOAS, ROM, or QF following intra-articular DF-HA injection. This finding suggests that the functional improvement resulting from the treatment investigated in this study (intra-articular administration of DF-HA) is closely associated with pain reduction. In other words, while the alleviation of knee joint pain appears to secondarily facilitate improvements in joint function, the functional benefits may be limited in cases where pain relief is not achieved.

A particularly intriguing finding is the significant increase in SLS time observed even in the Non-IM-G, where pain did not decrease. This could be attributed to the fact that SLS is an assessment influenced by a complex interplay of factors beyond just the presence or absence of pain, including QF, radiographic severity, lower limb alignment, and proprioception [18-20]. Furthermore, Bennell and Hinman [21] reported that experimentally induced acute knee pain did not significantly alter standing balance, failing to confirm the previously suggested correlation between pain intensity and balance scores. This suggests that balance deficits associated with chronic knee conditions like KOA may be more strongly influenced by factors other than the sensation of pain itself, such as long-term deterioration of joint function and abnormalities in neuromuscular control. Consequently, they concluded that treatment strategies focused primarily on pain reduction in knee joint disorders may not necessarily lead to improvements in balance ability.

The clinical implications of our findings suggest that while pain reduction may lead to some degree of temporary improvement in muscle strength and ROM, a more comprehensive approach involving longer-term rehabilitation, alignment correction, and proprioceptive training may be necessary to improve more complex motor functions, including balance ability. Furthermore, the slight but significant improvement in SLS time observed even in the Non-IM-G raises the possibility that the administered DF-HA formulation may have a minor effect on balance function through a mechanism independent of pain relief. However, given the small sample size of the Non-IM-G, this particular finding warrants cautious interpretation.

The limitations of this study include its retrospective design, the subjective nature of pain improvement assessment using VAS, and the small sample size of the Non-IM-G. The latter, in particular, may have resulted in insufficient statistical power. Additionally, the relatively short evaluation period of 3 to 7 days post-DF-HA administration limits our ability to assess long-term effects and changes in motor function. However, even considering these limitations, this study provides clinically valuable insights by suggesting that aggressive pain management in patients with advanced and previously difficult-to-treat KOA can lead to a tangible outcome of improved motor function, rather than just symptom relief.

## Conclusions

Aggressive pain management in refractory advanced KOA significantly and unequivocally improves key motor functions, including the JOAS, ROM, and QF. These findings strongly suggest that thorough pain control is a prerequisite for addressing the pathophysiology of chronic pain-induced motor function inhibition. However, the results also indicate that improving balance ability may necessitate a more comprehensive rehabilitation strategy in addition to pain relief. This study provides important insights into potential new treatment strategies for intractable knee joint pain. Future research should involve prospective studies with larger sample sizes, as well as investigations incorporating long-term changes in motor function and more detailed assessments of balance ability.
